# DeeplyEssential: a deep neural network for predicting essential genes in microbes

**DOI:** 10.1186/s12859-020-03688-y

**Published:** 2020-09-30

**Authors:** Md Abid Hasan, Stefano Lonardi

**Affiliations:** Department of Computer Science and Engineering, University of California Riverside, 900 University Ave, Riverside, 92507 CA USA

**Keywords:** Essential genes, Deep neural network, Microbes, Data leak

## Abstract

**Background:**

Essential genes are those genes that are critical for the survival of an organism. The prediction of essential genes in bacteria can provide targets for the design of novel antibiotic compounds or antimicrobial strategies.

**Results:**

We propose a deep neural network for predicting essential genes in microbes. Our architecture called DeeplyEssential makes minimal assumptions about the input data (i.e., it only uses gene primary sequence and the corresponding protein sequence) to carry out the prediction thus maximizing its practical application compared to existing predictors that require structural or topological features which might not be readily available. We also expose and study a hidden performance bias that effected previous classifiers. Extensive results show that DeeplyEssential outperform existing classifiers that either employ down-sampling to balance the training set or use clustering to exclude multiple copies of orthologous genes.

**Conclusion:**

Deep neural network architectures can efficiently predict whether a microbial gene is essential (or not) using only its sequence information.

## Background

Essential genes are those genes that are critical for the survival and reproduction of an organism [1]. Since the disruption of essential genes induces the death of an organism, the identification of essential genes can provide targets for new antimicrobial/antibiotic drugs [2, 3]. Essential genes are also critical for the creation of artificial self-sustainable living cells with a minimal genome [4]. Finally, essential genes have been a cornerstone in the study of the origin and evolution of organisms [5].

The identification of essential genes via wet-lab experiments is labor intensive, expensive and time-consuming. Such experimental procedures include single gene knock-out [6, 7], RNA interference, and transposon mutagenesis [8, 9]. Moreover, these experimental approaches can produce contradicting results [10]. With the recent advances in high-throughput sequencing technology, computational methods for predicting essential genes has become a reality. Some of the early prediction methods used comparative approaches by homology mapping, see, e.g., [11, 12]. With the introduction of large gene database such as DEG, CEG, and OGEE [13–15], researchers designed more complex prediction models using a wider set of features. These features can be broadly categorized into (i) sequence features, i.e., codon frequency, GC content, gene length [16–18], (ii) topological features, i.e., degree centrality, cluster coefficient [19–22], and (iii) functional features, i.e., homology, gene expression cellular localization, functional domain and other molecular properties [10, 23–26]. More recent studies about the 3D structure of proteins can also be incorporated in topological features set [27, 28].

Sequence-based features can be directly obtained from the primary DNA sequence of a gene and its corresponding protein sequence. Functional features such as network topology require knowledge of protein-protein interaction network, e.g., STRING and HumanNET [29, 30]. Gene expression and functional domain information can be obtained from databases like PROSITE and PFAM [31, 32]. Some of the less studied bacterial species, however, lack these functional and topological features, which would prevent the use of prediction tools that rely on them. Sequence-based classifiers are the most practical methods because they use the minimal amount of features.

Several studies have been published on the problem of predicting essential genes from their sequences. In [17], the authors developed a tool called ZUPLS that uses (i) a Z-curve derived from the sequence, (ii) homology mapping and (iii) domain enrichment score as features to predict essential genes in twelve prokaryotes after training the model on two bacteria. Although ZUPLS worked well on cross-organism prediction, the limited number of bacterial species used in the training set cast doubts on the ability of ZUPLS to generalize to more diverse bacterial species. In [33], the authors proposed a method that employs PCA on features derived from the gene sequence, protein domains, homologous and topological information. Among the studies that predict essential genes across multiple bacterial species, [26] employed several genomic, physio-chemical and subcellular localization features to predict gene essentiality across fourteen bacterial species. In their work, the authors dealt with the redundancy in the dataset (i.e., homologous genes shared by multiple bacterial genomes) by clustering genes based on their sequence similarities. In [16], nucleotide, di-nucleotide, codon, amino acid frequencies, and codon usage analysis were used for predicting essentiality in sixteen bacterial species. The authors used CD-HIT [34] for homology detection in both essential and non-essential genes. In [35], the authors identified essential genes in fifteen bacterial species using information theoretical features, e.g., Kullback-Leibler divergence between the distribution of *k*-mers (for *k*=1,2,3), conditional mutual information, and entropy. Although their work showed promising results for intra-organism and cross-organism predictions, the model performed rather poorly when trained on the complete bacterial dataset. Recently, [10] showed the most extensive prediction analysis of thirty-one bacterial species. The authors employed the features proposed in [26], with additional features such as transmembrane helices and Hurst exponent. Their algorithm used a regularized feature selection method called least absolute shrinkage and selection operator (Lasso) and used a support vector machine (SVM) as a classifier.

The most recent work on gene essentiality prediction [36] uses network-based features, Lasso for feature selection, and a Random Forest as the classifier. The authors used a recursive feature extraction technique to compute 267 features in three different categories i.e. *local features* such as degree distribution, *egonet features* which refers to the node and the induced subgraph formed by all of its neighbors, and *regional features* which are a combination of local and egonet features. They also used fourteen network centrality measures as a separate feature set for the essentiality prediction. Finally, they combined their network-based features with the sequence based features in [10] and [17] for their prediction model. For the models in [10, 36], and [17], the authors down-sampled non-essential genes to balance the training set but did not realize that their dataset contained multiple copies of homologous genes which created a “data leak” issue which biased their results (see below).

In this work we propose a feed-forward deep neural network (DNN) called DEEPLYESSENTIAL that uses features derived solely from the primary gene sequence, thus maximizing its practical application compared to other predictors that require structural or topological features which might not be readily available. To the best of our knowledge, this is the first time a deep neural network has been used for gene essentiality prediction.

## Materials and methods

### Dataset

Genomic data for thirty bacterial species were obtained from the database DEG, which is a curated and comprehensive repository of experimentally-determined bacterial and archaeal essential genes. Among the thirty bacterial species, nine are *Gram-positive* (GP) and twenty-one are *Gram-negative* (GN). DEG provides the primary DNA sequence and the corresponding protein sequence for both essential and non-essential genes, as well as gene functional annotations. We only considered protein-coding genes, i.e., we excluded RNA genes, pseudo-genes, and other non-coding genes. At the time of writing, DEG contained 28,876 essential protein-coding genes (of which 8746 belonged to a GP species and 20,130 belonged to a GN species) and 209,026 non-essential protein-coding genes (of which 45,002 were GP and 164,024 were GN). Table 1 shows the basic statistics of the dataset. Observe that the dataset is highly unbalanced. While species NC_000907 and NC_002771 have approximately the same number of essential and non-essential genes and bacteria NC_000908 has more essential genes than non-essential genes, for ten bacterial species less than 10% of their genes are essential. In order to improve the performance of our classifier, we balanced the dataset by down-sampling non-essential genes.

**Table 1:** The thirty bacterial species used for our experiments (GP is Gram-positive, GN is Gram-negative)

Accession	GP/GN	# Essential genes	# Non-essential genes
NC_000907	GN	1284	1024
NC_000908	GP	762	188
NC_000913	GN	1810	14000
NC_000915	GN	646	2270
NC_000962	GP	4144	17586
NC_000964	GP	542	7808
NC_002163	GN	788	5602
NC_002505/002506	GN	1558	5886
NC_002516	GN	906	21266
NC_002745	GP	604	4562
NC_002771	GP	620	644
NC_003197	GN	460	8456
NC_004347	GN	804	2206
NC_004631	GN	1422	15822
NC_004663	GN	650	8906
NC_005966	GN	998	5188
NC_006351/006350	GN	1010	10444
NC_007297	GP	454	2674
NC_007795	GP	702	5082
NC_008463	GN	670	1920
NC_008601	GN	784	2658
NC_009009	GP	436	4104
NC_009511	GN	1070	8630
NC_010729	GN	1488	6870
NC_011375	GP	482	2354
NC_011916	GN	960	6448
NC_016776	GN	1094	7486
NC_016810	GN	706	8070
NC_016856	GN	210	10420
NC_007650/007651	GN	812	10452

### Feature selection

As said, various intrinsic gene features, such as protein domains, protein-interaction network data, etc. have been used for predicting gene essentiality [33, 35]. DEEPLYESSENTIAL utilizes codon frequency, maximum relative synonymous codon usage (RSCU), codon adaptation index (CAI), gene length and GC content. In addition to DNA-derived features, DEEPLYESSENTIAL uses amino acid frequencies and the length of the protein sequence.

#### Codon frequency

**Fig. 1:**
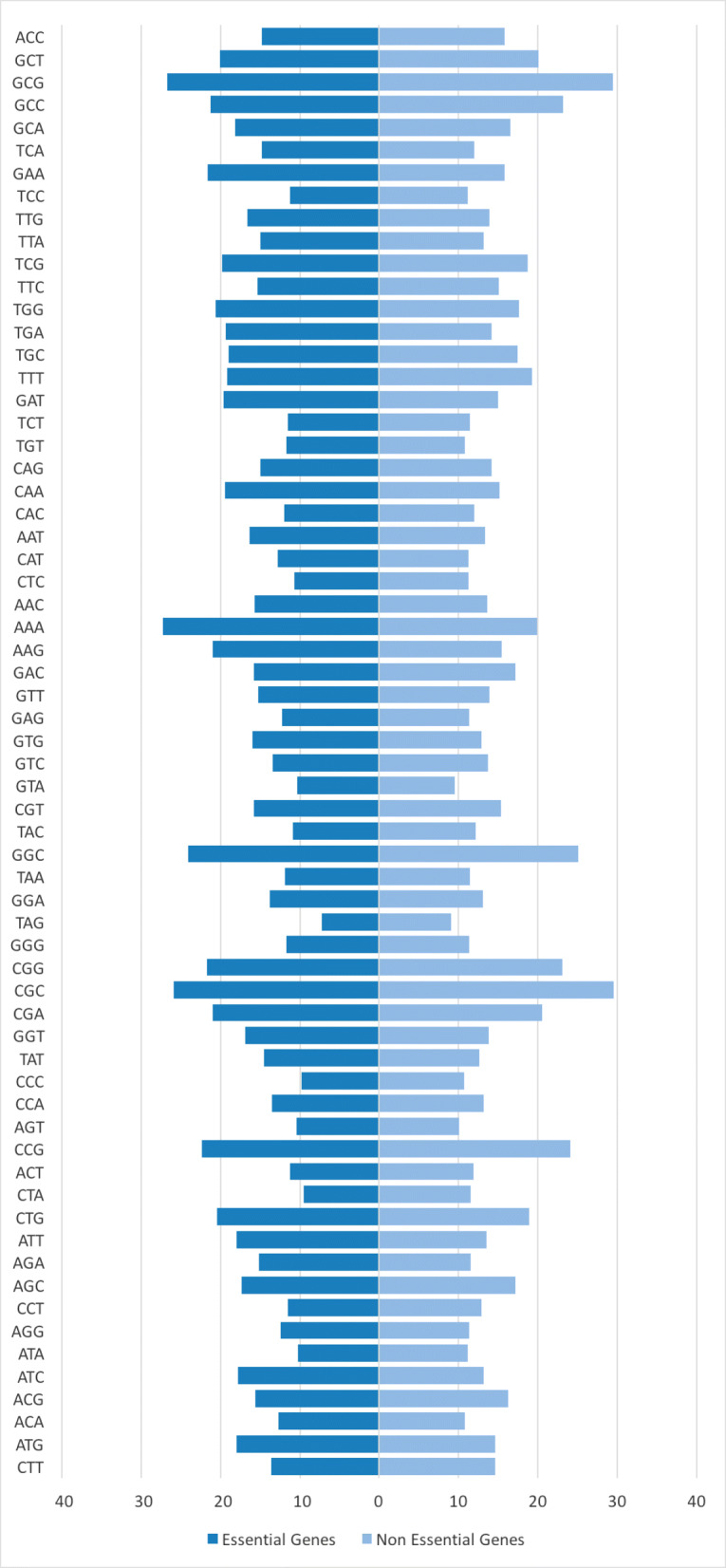
Normalized codon frequency of genes in GP + GN dataset

Codon frequency has been recognized as an important feature for gene essentiality prediction [10, 26]. Given the primary DNA sequence of a gene, its codon frequency is computed by sliding a window of three nucleotides along the gene. The raw count of 4^3^=64 codons is then normalized by the total number of genes. Observe in Fig. 1 that the codon frequency is significantly different in the two classes. For instance, codon AAA, GAA, TGA, GAT, AAG, ATT and AGA have at least 30% difference in their normalized codon frequency between essential and non-essential genes.

#### Gene length and GC content

**Fig. 2:**
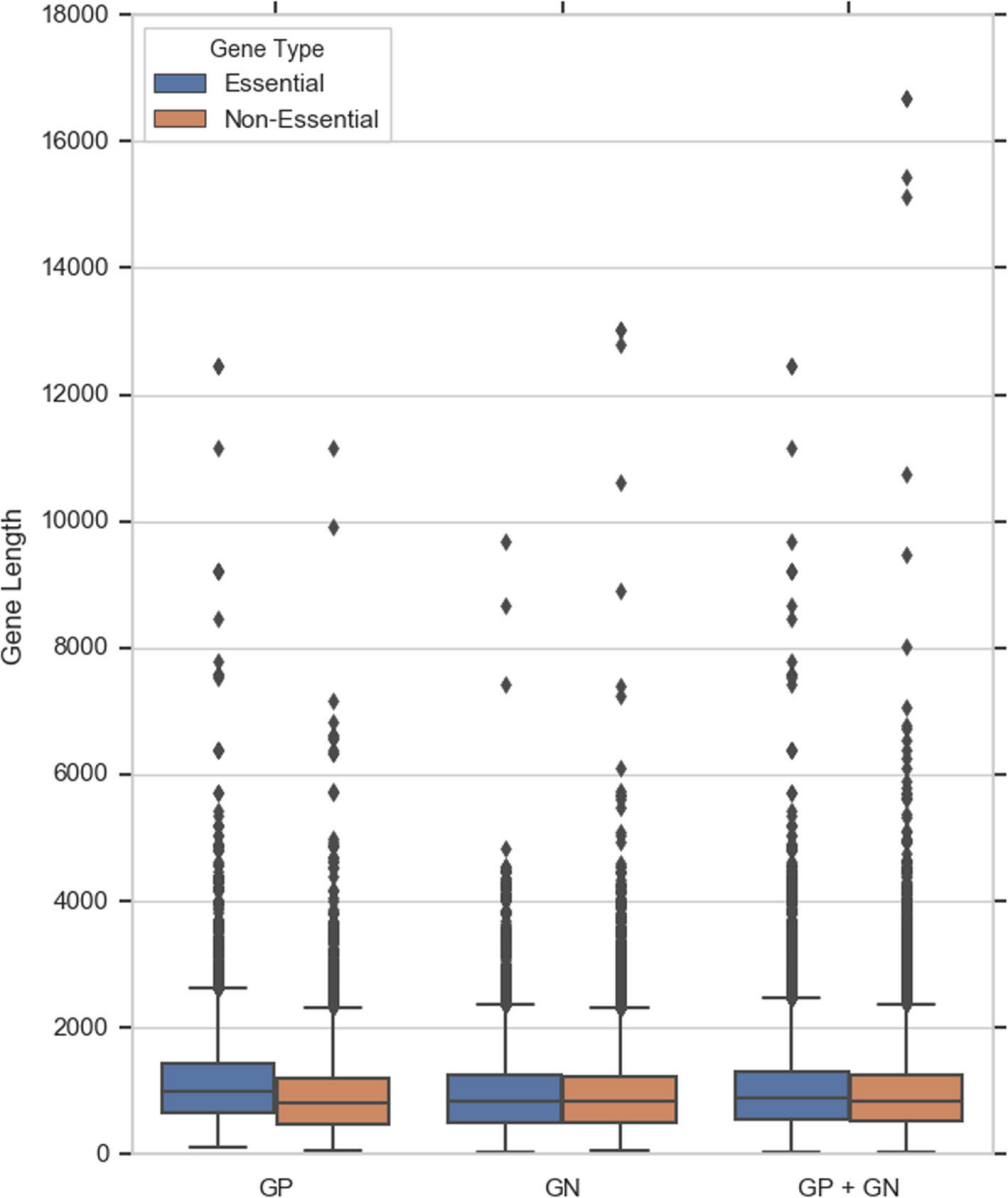
Distribution of gene lengths in datasets GP+GN, GN, GN

**Fig. 3:**
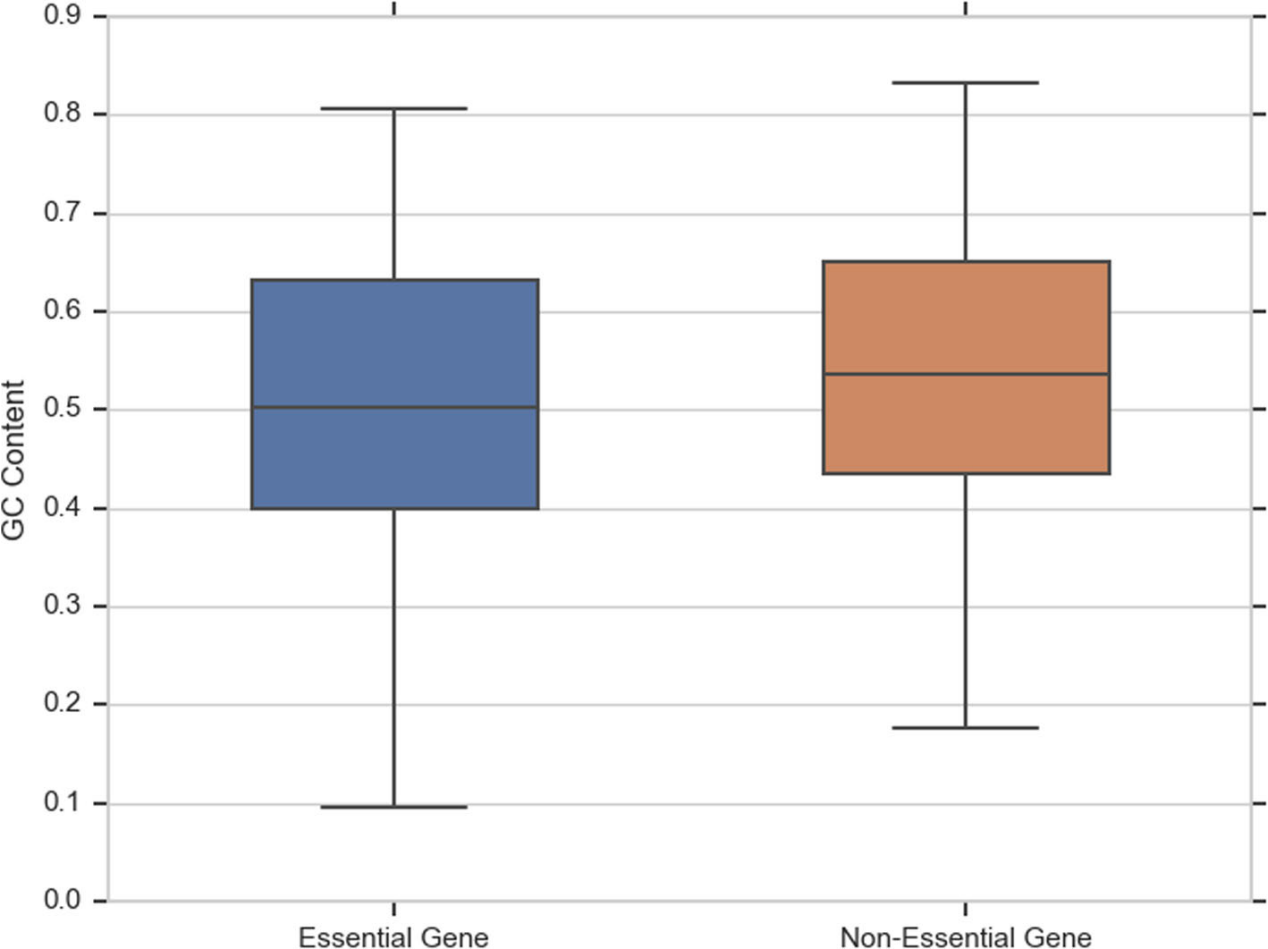
GC content distribution in essential and non-essential gene sets in the GP+GN dataset

Other distinguishing features for gene essentiality are gene length and GC content. Figure 2 shows the distribution of gene length in GP, GN and the combined dataset (GP+GN). Observe that genes in the GP+GN dataset and the GN dataset have a similar average length in the two classes, while essential genes in the GP dataset are on average longer than non-essential genes. As said, the GC content is another informative feature of essentiality prediction. Figure 3 shows the difference in distribution in GC content between the two classes. Observe that non-essential genes have higher GC content than essential genes.

#### Relative synonymous codon usage

Unbalanced synonymous codon usage is prevalent both in prokaryotes and eukaryotes [37]. The degree of bias varies among genes not only in different species but also among genes in the same species. Differences in codon usage in one gene compared to its surrounding genes may imply its foreign origin, different functional constraints or a different regional mutation. As a result, examining codon usage helps to detect changes in evolutionary forces between genomes. Essential genes are critical for the survival of an organism thus codon usage acts as a strong distinguishing feature. To calculate the relative synonymous codon usage we compare the observed number of occurrence of each codon to the expected number of occurrences (assuming that all synonymous codons have equal probability). Given a synonymous codon *i* that has an *n*-fold degenerate amino acid, we compute the *relative synonymous codon usage* (RSCU) as follows

$$\text{RSCU}_{i} = \frac{X_{i}}{(1/n)\sum_{{i=1}}^{n} X_{i}} $$

where *X*_*i*_ is the number of occurrence of codon *i*, and *n* is 1, 2, 3, 4, or 6 (according to the genetic code).

#### Codon adaptation index

The *codon adaptation index* (CAI) estimates the bias towards certain codons that are more common in highly expressed genes [37]. The CAI is defined as the geometric mean of the relative adaptedness statistics. The *relative adaptedness* for codon *i* is defined on the relative frequency of the codon in a species-specific reference set of highly expressed genes. Formally, the relative adaptedness is defined by

$$r_{i} = \frac{\text{RSCU}_{i}}{\text{RSCU}_{max}} = \frac{X_{i}}{X_{max}} $$

where RSCU_*max*_ and *X*_*max*_ are corresponding RSCU and *X* value of the most frequently used codon. The CAI for a gene is defined by

$$\text{CAI} = \left (\prod_{i=1}^{L}r_{i} \right)^{\frac{1}{L}} $$

where *L* is the number of codons in the gene excluding methionine, tryptophan, and stop codon. The range of CAI is (0,1] where higher values indicating a higher proportion of the most abundant codons.

#### Protein sequence features

Another informative set of features used for the prediction of gene essentiality are those derived from the corresponding protein sequences. Previous studies have used frequencies of rare amino acids, and the number of codons that are one-third base mutations removed from the stop codons [10]. DEEPLYESSENTIAL only uses amino acids frequencies and the lengths of the protein sequences.

#### Combining all the features

Given the primary DNA sequence of a gene, we generate 4^3^=64 values for the codon frequency, and single values for the GC content, gene length, CAI and *RSCU*_*max*_. From the protein sequence, we compute the amino acid frequency vector (20 values), and one value for the protein length. The total number of features used by DEEPLYESSENTIAL is 89.

### Multi-layer perceptron

A multi-layer perceptron (MLP) consists of multiple layers of computational units where the information flows in the forward direction, from input nodes through hidden nodes to the output nodes without any cycles [38]. MLP networks have been used successfully for several molecular biology problems, see, e.g. [39–41]. The architecture of DEEPLYESSENTIAL is composed of an input layer, multiple hidden layers, and an output layer. The output layer encodes the probability of a gene to be essential. The addition of a dropout layer makes the network less sensitive to noise during training and increase its ability to generalize. This layer randomly assigns zero weights to a fraction of the neurons in the network [42].

Let $\overrightarrow {x} = (x_{1}, \cdots, x_{n})^{T}$ be the input to the MLP. Let vector *y* denote the output of the *i*^th^ hidden layer. The output *y*^*i*^ depends on the input in the previous layer as follows

$$y^{i} = a\left(W^{i}x^{(i-1)} + b^{(i-1)}\right) $$

where *a* is the activation function, *b* is the bias and *W* is the weight matrix for the edges in the network. During the training phase, the network learns the weights *W* and the bias *b*. DEEPLYESSENTIAL uses a rectified linear unit (ReLU) in each neuron in the hidden layers. ReLU is an element-wise operation that clamps all negative values to zero.

In the output layer DEEPLYESSENTIAL uses a sigmoid as the activation function to perform discrete classification

$$y = \frac{1}{1 + e^{-x}} $$

**Fig. 4:**
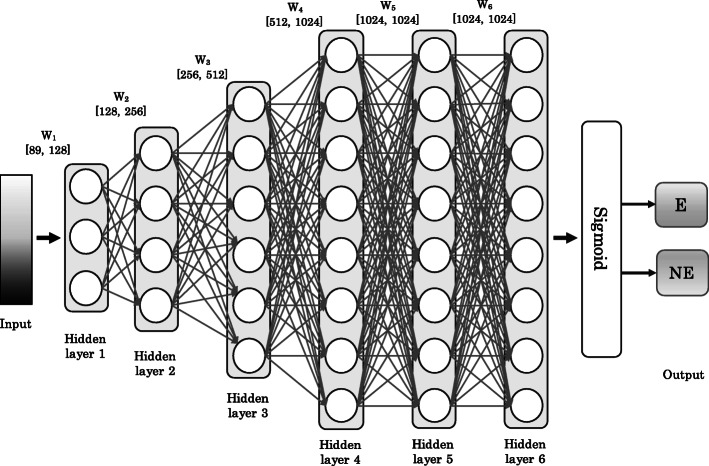
The architecture of the neural network used in DEEPLYESSENTIAL

The loss function is the binary cross-entropy defined by

$$\sum_{c = 1}^{M} \hat{y}_{o, c} \log(p_{o,c}) $$

where *M* is the number of classes (two in our case), $\hat {y}$ is the binary indicator if class label *c* is the correct classification for observation *o*, and *p* is the predicted probability that observation *o* belongs to class *c*. Figure 4 illustrates the architecture of the neural network used in DEEPLYESSENTIAL.

## Results and discussion

### Classifier design and evaluation

As mentioned above, the number of non-essential genes is significantly larger than the number of essential genes. To address this imbalance in the training set and allow for unbiased learning, we randomly down-sample non-essential genes. In [18], the authors showed that balancing the dataset did not negatively influence the prediction of gene essentiality.

#### Model hyper-parameters

Recall that each gene (and its corresponding protein) is represented by 89 features in the input layer. The architecture of DEEPLYESSENTIAL was determined by running extensive experiments on the training data over a wide range of hyper-parameters. The number of hidden layers, the number of nodes in each of the hidden layers, the batch size, the dropout rate and the type of optimizer were selected by optimizing the performance of the classifier during cross-validation. Table 2 lists the range of hyper-parameters considered and the values of the hyper-parameters selected for the final architecture of DEEPLYESSENTIAL.

**Table 2:** Hyperparameters for DEEPLYESSENTIAL

Parameters	Range	Selected parameter
# hidden layers	[2 - 8]	6
# nodes	[32, 64, 128, 512, 1024, 2048]	128, 256, 512, 1024, 1024, 1024
Dropout rate	[0.1 - 0.5]	0.3
Epochs	–	100 (early stopping)
Optimizer	sgd, adam, adadelta, RMSProp	adadelta

Observe in Fig. 4 that the final fully-connected layer reduces the 1024 dimensional vector to a two-dimensional vector corresponding to the two prediction classes (essential/non-essential). The sigmoid activation function forces the output of the two neurons in the output layer to sum to one. Thus the output value represents the probability of each class. Among the available optimizer in Table 2, we chose adadelta because of its superior performance. Adadelta is parameter-free, thus we did not need to define the learning rate. The training was run for 100 epochs with early stopping criteria.

We trained DEEPLYESSENTIAL on three datasets, namely GP, GN, and GP+GN. For each dataset, 80% data is used for training, 10% data for validation and 10% data for testing. The random selection was repeated ten times, i.e., ten-fold cross-validation was performed to complete the inference.

#### Evaluation metrics

The tools described in [10, 16, 26] and [35] are currently unavailable. We ran DEEPLYESSENTIAL on the datasets used in the corresponding papers, and compared DEEPLYESSENTIAL’s classification metrics to the published metrics.

We evaluated the performance of DEEPLYESSENTIAL using the Area Under the Curve (AUC) of the Receiver Operating characteristic Curve (ROC). ROC plot represents the trade-off between sensitivity and specificity for all possible thresholds. Although our primary evaluation measure is the AUC score, we also report the following additional performance measures

$$\begin{array}{@{}rcl@{}} \mathrm{Sensitivity (Sn)} & = & \frac{TP}{(TP + FN)} \\ \mathrm{Specificity (Sp)} & = & \frac{TN}{(FP + TN)} \\ \text{PPV} & = & \frac{TP}{(TP + FP)} \\ \text{Accuracy} & = & \frac{(TP + TN)}{(TP + FN + TN + FP)} \end{array} $$

where *TP*, *TN*, *FP* and *FN* represent the number of true positives, true negatives, false positives, and false negatives, respectively.

All experiments were carried out a Titan GTX 1080 Ti GPU, running Keras v2.1.5.

### Gene essentiality prediction

We collected essential and non-essential gene for thirty bacterial species described above into three datasets, namely GP, GN, and GP+GN. After re-balancing the dataset by down-sampling non-essential genes, we extracted the features for each gene as explained above. Table 3 shows the basic statistics for each dataset.

**Table 3:** Basic statistics for GP, GN, and GP+GN (balanced and unbalanced)

Dataset	# Training samples	# Validation samples	# Test samples
GP	7,065	883	884
GN	14,364	1,795	1,797
GP+GN (bal)	21,432	2,678	2,680
GP+GN (unbal)	90,571	11,321	11,322

**Fig. 5:**
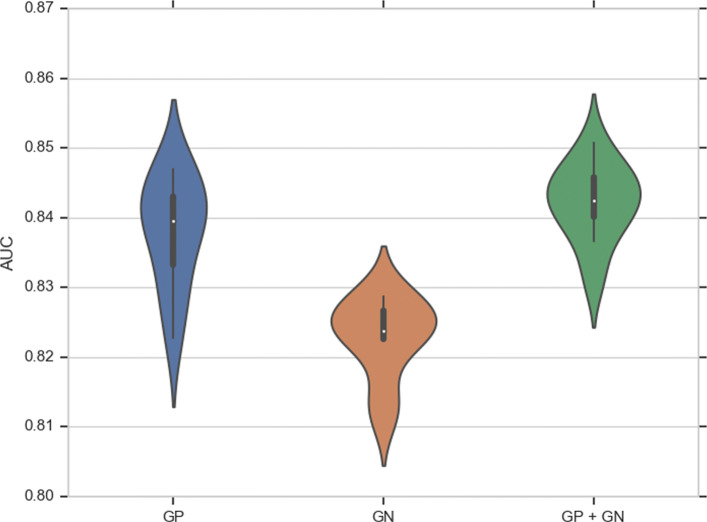
Violin plot of DEEPLYESSENTIAL’s AUC across ten experiments on the GP, GN, GP+GN datasets

**Fig. 6:**
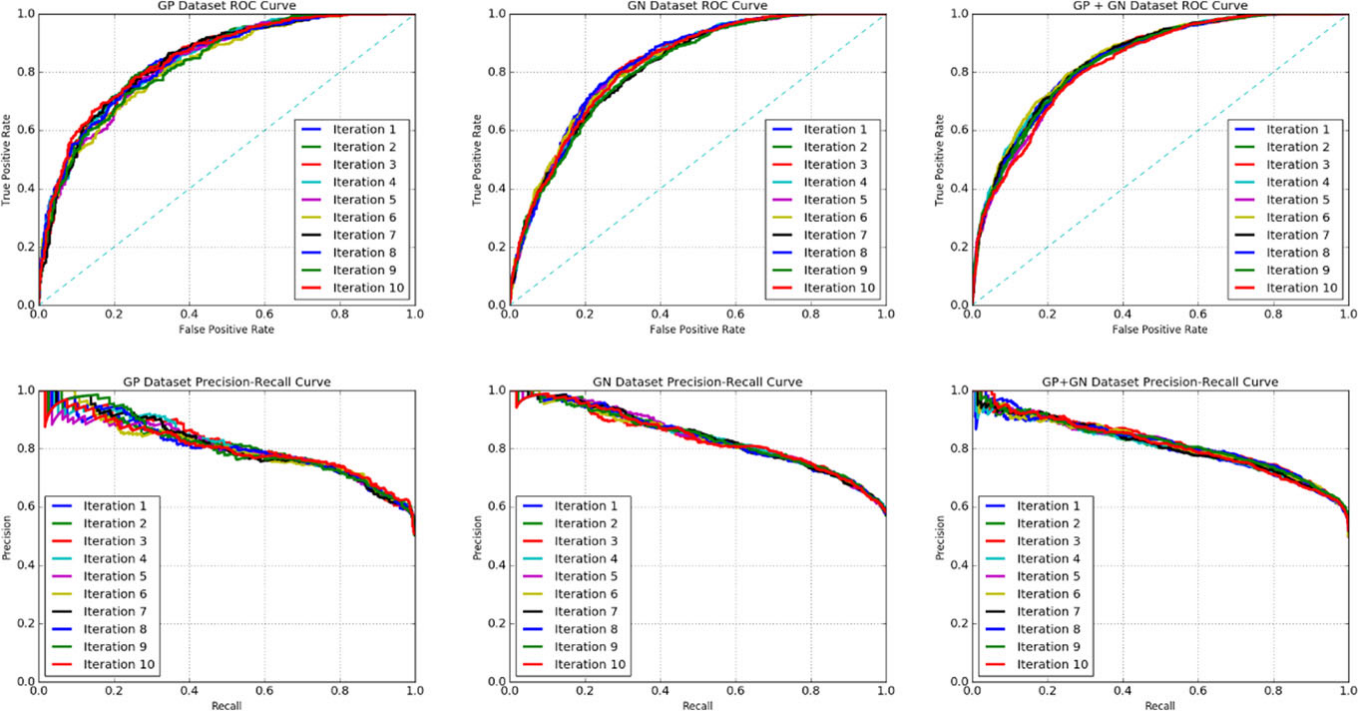
DEEPLYESSENTIAL’s ROC and AUPR curves on GP, GN, GP+GN

Table 4 shows the training classification performance of DEEPLYESSENTIAL, averaged over ten repetitions. The violin plot in Fig. 5 shows the distribution of AUCs across the ten repetitions of the experiment, which appears very stable. The receiver operator curves (ROC) are shown in Fig. 6. DEEPLYESSENTIAL yielded an area under the curve of 0.838, 0.829 and 0.842 for GP, GN, and GP+GN on average, respectively. The ROC curve also indicates the relation between the number of training samples and the stability of the prediction performance. Observe that DEEPLYESSENTIAL’s performance was more stable on the GP+GN dataset than the GP dataset (which contains the smallest number of samples). Tthe precision-recall curves shows the ability of DEEPLYESSENTIAL to yield low false positive rate and low false negative rate consistently across all datasets.

**Table 4:** Training classification performance of DEEPLYESSENTIAL on GP, GN, GP+GN

Metric	GP	GN	GP+GN
AUC	0.838	0.823	0.842
Sensitivity	0.741	0.784	0.801
Specificity	0.758	0.708	0.721
PPV	0.774	0.722	0.749
Accuracy	0.749	0.745	0.762

### Comparison with down-sampling methods

**Fig. 7:**
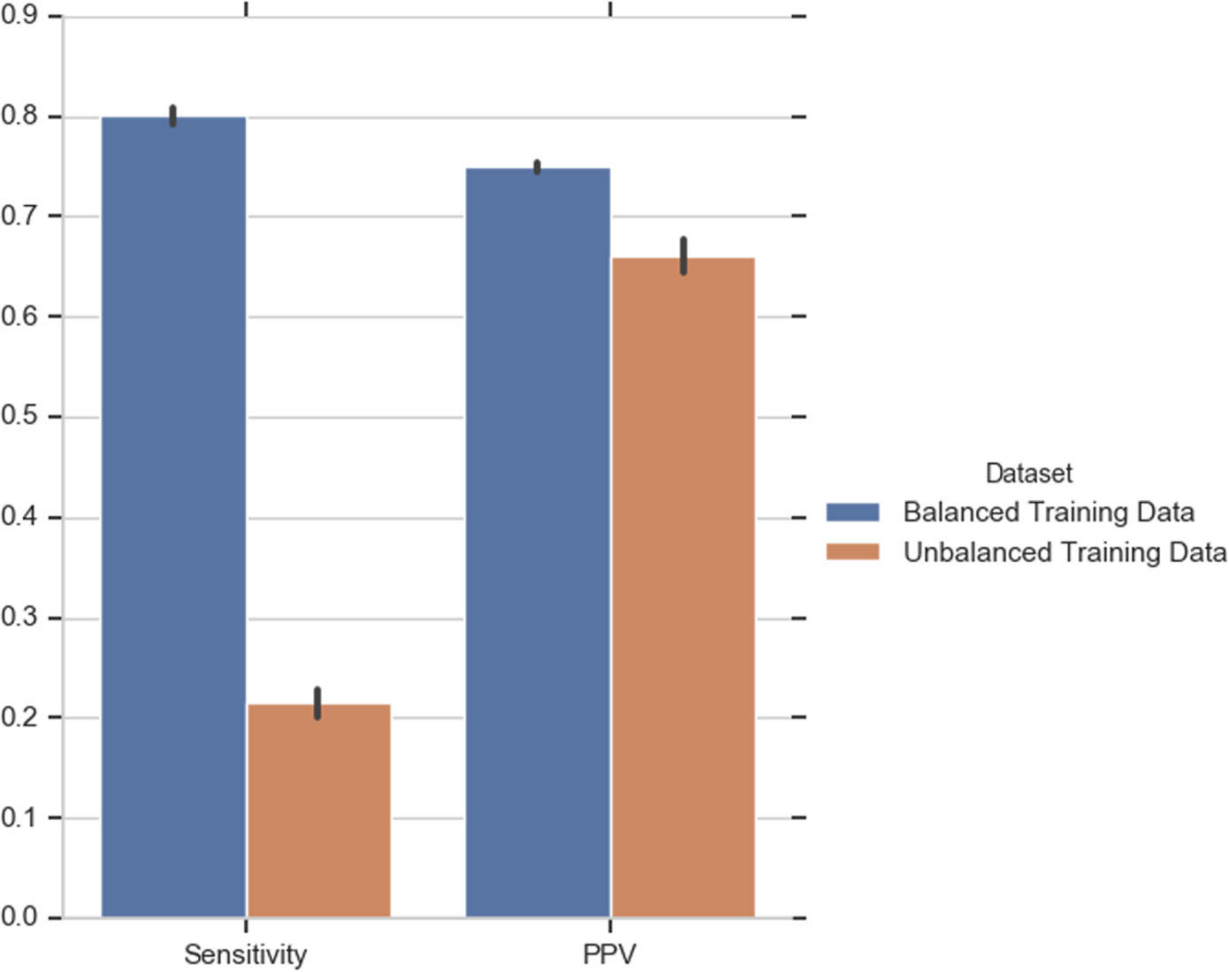
Comparing the prediction performance of DEEPLYESSENTIAL when trained on balanced or unbalanced GP+GN dataset

As mentioned in the previous section, the gene essentiality dataset is highly unbalanced. It is well-known that class imbalance can negatively affect the performance of a classifier [43]. To quantify how class imbalance affects the performance of our classifier we trained DEEPLYESSENTIAL on the full (unbalanced) dataset that has 322.6% more non-essential genes than essential genes. Figure 7 shows that the sensitivity and Positive Predictive Value (PPV) of the classifier trained on unbalanced data are much worse than the balanced dataset. As said, some of the existing methods use down-sampling to address this problem. Both Liu et al. [10] and Azhagesan et al. [36] randomly down-sampled the majority class data to match the size of the minority class. DEEPLYESSENTIAL also uses this approach. Table 5 shows the performance DEEPLYESSENTIAL compared to the two published methods that use down-sampling. Observe that DEEPLYESSENTIAL achieves the best AUC, sensitivity, and PPV.

**Table 5:** Comparing the performance of DEEPLYESSENTIAL on down-sampled dataset against methods that solely use sequence features; numbers in boldface indicate the best performance

Method	# features	AUC	Sensitivity	PPV
Liu et al. 2017	40	0.794	0.715	0.243
Azhagesan et al. 2018	267	0.838	0.754	0.321
ZUPLS	274	0.705	0.663	0.255
DeeplyEssential	89	**0.842**	**0.801**	**0.749**

### Identification of “data leak” in the gene essentiality prediction

Bacteria are unicellular organisms with a relatively small set of genes. Across bacterial species, a significant fraction of the genes is conserved because they perform similar fundamental biological functions. These conserved genes are quite similar at the sequence level. All published methods rely on a dataset containing multiple bacteria on which genes have been labeled essential or non-essential. Let us call *x* and *y* two homologous genes, i.e., two genes that have a very similar primary DNA sequence. If *x* is used on the training and *y* if used for testing, this introduces a bias, or a “data leak”. Training examples and testing examples are supposed to be distinct, and in this hypothetical scenario, they are not.

To quantify the effect of the data leak issue, we clustered the set of all genes across the thirty bacterial species using OrthoMCL [44]. OrthoMCL is a popular method for clustering orthologous, homologous and paralog proteins which use reciprocal best hit alignment to detect potential in-paralog/recent paralog pair, and reciprocal best hit alignments between two genomes to identify potential ortholog pairs. A similarity graph is then generated based on the proteins that are interlinked. To split large clusters, a Markov Clustering algorithm (MCL) is then invoked [45]. Inside MCL clusters, weights between each pair of proteins are normalized to correct for evolutionary differences.

**Fig. 8:**
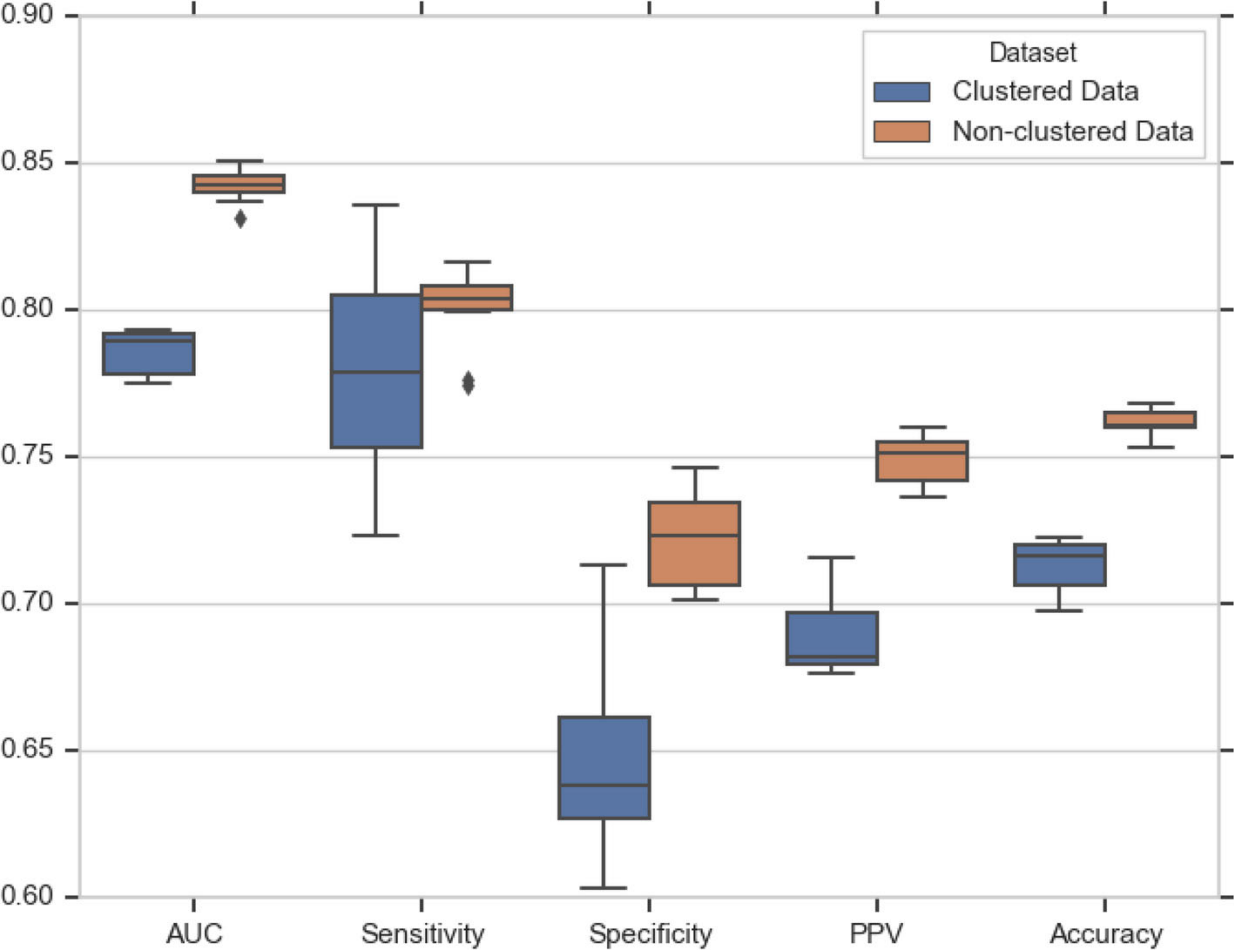
Effect of “data leak” on DEEPLYESSENTIAL’s prediction performance on GP+GN dataset

As said, OrthoMCL produces a list of clusters where each cluster consists of genes that have been determined to be orthologous. To quantify the effect of gene sequence similarity on the prediction performance, we created a dataset where no gene from a single cluster can be assigned to both training set and testing set. The modified dataset contains 11,168 training samples, 2,798 validation samples, and 4,270 testing samples. The prediction was repeated ten times. Table 6 shows how the clustering step heavily influences DEEPLYESSENTIAL’s prediction performance. AUC decreased by more than 7% (on average), while the accuracy decreased by 6.9% (along with a significant decrease in all performance measures). Figure 8 shows the difference in performance before and after clustering. While the AUCs were stable across experiments, sensitivity, specificity, and PPV varied largely across experiments on the clustered dataset.

**Table 6:** Comparing the effect of clustering on the prediction performance of DEEPLYESSENTIAL on the GP+GN dataset

Metric	Non-clustered	Clustered	Difference (%)
AUC	0.842	0.786	7.12%
Sensitivity	0.801	0.780	2.69%
Specificity	0.721	0.646	11.60%
PPV	0.749	0.688	8.86%
Accuracy	0.762	0.713	6.87%

### Comparison with methods that cluster orthologous genes

Some published studies have addressed the data leak issue by identifying homologous genes using sequence similarity metrics. In [35], the authors used the Kullback-Leibler divergence to measure the distance between *k*-mer distribution (for *k*=1,2,3) obtained from sequences. In [16], the authors used CD-HIT to remove redundancy in the training data and improve the generalization ability of their model. As explained in the previous section, DEEPLYESSENTIAL uses OrthoMCL to cluster homologous genes to prevent similar genes to appear in both training and testing dataset. Table 7 shows the performance comparison of DEEPLYESSENTIAL with [16] and [35] on their respective datasets.

**Table 7:** Comparing the performance of DEEPLYESSENTIAL and Ning et al. and Nigatu et al. on their respective datasets [16, 35]; numbers in boldface indicate the best performance

Method	Clustering method	AUC
Ning et al. 2014	CD-HIT	0.758
DeeplyEssential	OrthoMCL	**0.818**
Nigatu et al. 2017	*Kullback-Leibler divergence*	0.650
DeeplyEssential	OrthoMCL	**0.840**

Observe that in both cases DEEPLYESSENTIAL achieves the best predictive performance. As said, although each of these two approaches uses a distinct method to determine orthologous genes, the use of the same dataset for the experiments ensures a fair comparison.

### Feature importance

**Fig. 9:**
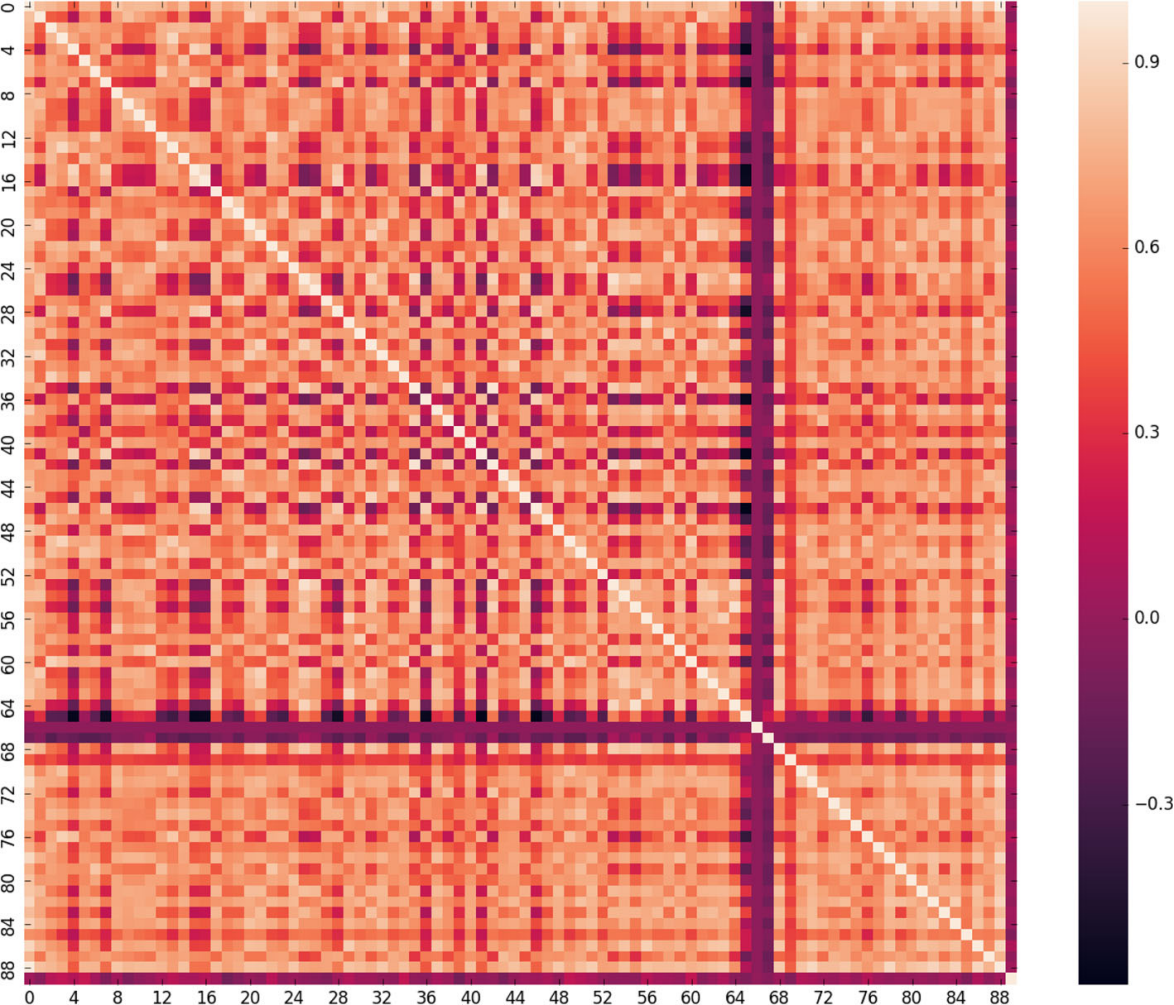
Pairwise correlation among all features; features 0–65 are DNA specific feature; features 68–89 are protein specific features

**Fig. 10:**
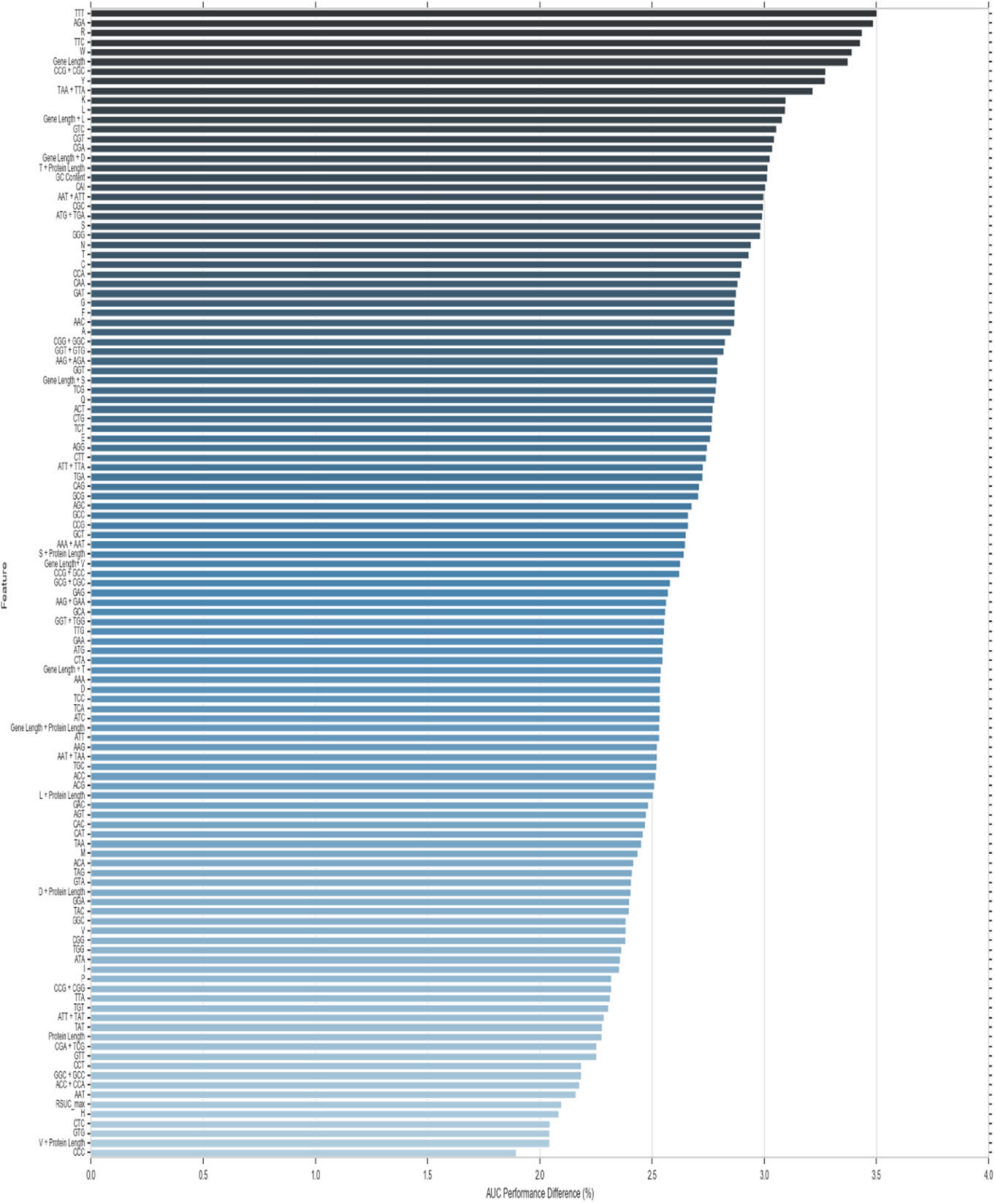
Changes in AUC predictive performance due to the removal of a feature or pairs of correlated features

DEEPLYESSENTIAL uses exclusively sequence-based features and yet produces higher prediction performance. Unlike other machine learning classifiers, the DNN architecture does not readily provide any insight about the feature set that contributed maximally towards the prediction performance. To understand the impact of a feature on the predictive performance, we carried out an ablation study which removes one or more features from the input and determines the performance difference. In order for the ablation study to be informative, features cannot be highly correlated. In this latter case, the removal of a feature is immediately compensated by its highly correlated feature. To address this issue, we first computed the pairwise Pearson correlation among all input features. Figure 9 illustrate the heatmap of the pairwise correlation. Each axis shows the indices of the features: indexes 0–65 contains DNA specific feature, index 68–89 contains protein specific features. GC content, CAI and RSCU_*max*_ have a negative correlation with all other features. There were nineteen pair of features showing a correlation higher than 0.9 (in absolute value). For the ablation study, we either removed one feature at a time (if uncorrelated) or one of the 19 feature pairs. We tested the performance changes on the GP + GN dataset using 5-fold cross-validation. Specifically, we measured the difference in AUC and ordered the features based on their impact in decreasing the predictive performance (Fig. 10). Observe that codon TTT caused the highest AUC decrease (3.5%) while AGA, TTC, CGT, CGA, gene length, protein length, GC content, CAI, amino acids K, L, R, W, Y, C, G, E, F, and pairs of correlated features CCG+CGC, TAA+TTA, gene length+L, D, and protein length+T induced 2.5% –3% AUC decrease. Our finding that gene and protein length are highly informative features for essentiality prediction recapitulate their prominence, as illustrated in other sequence-based methods, e.g., [10] and [17]. Moreover, it is well-known that in essential genes within the functional category related to information storage and process, encoded amino acids K, L and subcategories of encoded amino acids C, G, E, F are preferentially suited at the leading strand where these are responsible for energy production and conversion, carbohydrate transport and other essential metabolic processes [46].

### Discussion

A large number of structural and functional features have been used for gene essentiality prediction, i.e. producibility, choke points, load scores, damages, degree of centrality, clustering coefficient, closeness centrality, betweenness centrality, gene expression, phyletic retention, among others. These features cannot be obtained from the gene sequences and are often not available for many bacterial species. To maximize its practical utility, DEEPLYESSENTIAL uses exclusively features derived directly from the sequence.

Previous works have addressed the high imbalance of the training dataset by either down-sampling non-essential genes or by clustering orthologous genes across species. In order to make a meaningful and fair comparison, we compared DEEPLYESSENTIAL’s performance to both approaches. In fact, our experiments showed that DEEPLYESSENTIAL has better predictive performance both on down-sampled and clustered datasets. On the down-sampled dataset used in [10], DEEPLYESSENTIAL demonstrated an improvement of 12.8% in AUC compared to [10]. In addition, DEEPLYESSENTIAL produced significantly better sensitivity and precision than the three methods in Table 5, achieving 6.2% improved sensitivity and 137.4% improved precision compared to [36]. If one uses all the 597 features in [36], then this latter method achieves 1.7% improved AUC compared to DEEPLYESSENTIAL. We believe that collecting this very large amount of features from multiple databases does not warrant the additional (minor) benefit in predictive performance. DEEPLYESSENTIAL also achieved better performance on clustered datasets. Table 7 shows 7.9% and 29.2% improved AUC compared to [16] and [35], respectively.

As an alternative to the proposed approach that uses a carefully selected set of features as input, one could consider training a convolutional neural network (CNN) that uses exclusively one hot encoding of the DNA and protein sequence as input. One hot encoding is a process that converts categorical variables into a numerical vector that is convenient for the prediction by machine learning models. We expect that the limited size of the available training data would be insufficient to allow for the CNN to extract relevant features. As a consequence, we expect CNN-based classifiers not to be as accurate compared to the architecture proposed here.

## Conclusion

We proposed a deep neural network architecture called DEEPLYESSENTIAL to predict gene essentiality in microbes. DEEPLYESSENTIAL makes a minimal assumption about the input data (i.e, it only uses the gene sequence), thus maximizing its practical application compared to other predictors that require structural or topological features which might not be readily available. Extensive experiments show that DEEPLYESSENTIAL has better predictive performance than existing prediction tools. We believe that DEEPLYESSENTIAL could be further improved if more annotated bacterial data was available, making it an essential tool for drug discovery and synthetic biology experiments in microbes.

## Data Availability

The code of DeeplyEssential is freely available at https://github.com/ucrbioinfo/DeeplyEssential.

## Abbreviations

DEG: Database of essential genes
PCA: Principal component analysis
GP: Gram positive
GN: Gram negative
DNN: Deep neural network
RSCU: Relative synonymous codon usage
CAI: Codon adaptation index
AUC: Area under the curve
ROC: Receiver operator curve
PPV: Positive predictive value
CNN: Convolutional neural network
